# Endothelial Monocyte-Activating Polypeptide-II Induces BNIP3-Mediated Mitophagy to Enhance Temozolomide Cytotoxicity of Glioma Stem Cells via Down-Regulating MiR-24-3p

**DOI:** 10.3389/fnmol.2018.00092

**Published:** 2018-03-26

**Authors:** Jian Zhang, Libo Liu, Yixue Xue, Yawen Ma, Xiaobai Liu, Zhen Li, Zhiqing Li, Yunhui Liu

**Affiliations:** ^1^Department of Neurosurgery, Shengjing Hospital of China Medical University, Shenyang, China; ^2^Liaoning Research Center for Translational Medicine in Nervous System Disease, Shenyang, China; ^3^Department of Neurobiology, College of Basic Medicine, China Medical University, Shenyang, China; ^4^Key Laboratory of Cell Biology, Ministry of Public Health of China, and Key Laboratory of Medical Cell Biology, Ministry of Education of China, Shenyang, China

**Keywords:** GSCs, EMAP-II, TMZ, BNIP3, mitophagy

## Abstract

Preliminary studies have shown that endothelial-monocyte-activating polypeptide-II (EMAP-II) and temozolomide (TMZ) alone can exert cytotoxic effects on glioma cells. This study explored whether EMAP-II can enhance the cytotoxic effects of TMZ on glioma stem cells (GSCs) and the possible mechanisms associated with Bcl-2/adenovirus E1B 19 kDa protein-interacting protein 3 (BNIP3)-mediated mitophagy facilitated by miR-24-3p regulation. The combination of TMZ and EMAP-II significantly inhibited GSCs viability, migration, and invasion, resulting in upregulation of the autophagy biomarker microtubule-associated protein one light chain 3 (LC3)-II/I but down-regulation of the proteins P62, TOMM 20 and CYPD, changes indicative of the occurrence of mitophagy. BNIP3 expression increased significantly in GSCs after treatment with the combination of TMZ and EMAP-II. BNIP3 overexpression strengthened the cytotoxic effects of EMAP-II and TMZ by inducing mitophagy. The combination of EMAP-II and TMZ decreased the expression of miR-24-3p, whose target gene was BNIP3. MiR-24-3p inhibited mitophagy and promoted proliferation, migration and invasion by down-regulating BNIP3 in GSCs. Furthermore, nude mice subjected to miR-24-3p silencing combined with EMAP-II and TMZ treatment displayed the smallest tumors and the longest survival rate. According to the above results, we concluded that EMAP-II enhanced the cytotoxic effects of TMZ on GSCs' proliferation, migration and invasion both *in vitro* and *in vivo*.

## Introduction

Glioma is the most common primary invasive tumor of the central nervous system (Ostrom et al., 2015; Louis et al., 2016). The National Comprehensive Cancer Network (NCCN) Clinical Practice Guidelines state that the current standard of care with respect to glioma management includes surgical resection followed by radiotherapy with concomitant and adjuvant chemotherapy with temozolomide (TMZ) (Stupp et al., 2005; Nabors, 2016). The anti-cancer effects of TMZ, an oral second-generation alkylating agent, are facilitated by its rapid hydrolysis into 5-(3-methyltriazen-1yl) imidazole-4-carboxamide (MITC) under physiological pH conditions (Pace et al., 2003). TMZ has been shown to confer more survival benefits than other chemotherapy drugs. However, the European Organization for Research and Treatment of Cancer/National Cancer Information Center (EORTC/NCIC) trial shows that the median survival time of patients treated with TMZ was 19 months, while the median survival time of patients treated without TMZ was 14.6 months (Rapp et al., 2015). Glioma stem cells (GSCs) are individual glioma founder cells with the capacity for infinite self-renewal and long-term proliferation, as well as the capacity for neurosphere formation and multipotential differentiation (Bao et al., 2006). It has been shown that GSCs is more resistant to conventional therapies than its differentiated counterpart. It has also proved that functional and some mechanistic evidence of the conversion of non-GSCs into GSCs both *in vitro* and *in vivo* after exposure to TMZ. Therefore One of the most significant impediments to the success of glioma chemotherapy is the presence of GSCs (Auffinger et al., 2014).

Endothelial-monocyte-activating polypeptide-II (EMAP-II) is a 22-kDa secretory polypeptide protein that was originally isolated from murine methylcholanthrene A-induced fibrosarcoma. It inhibits tumor angiogenesis and causes tumor cell apoptosis without effecting normal cells and thus has been applied in cancer therapy (Berger et al., 2000a,b). EMAP-II-induced autophagy has been shown to kill glioma cells and GSCs (Ma et al., 2015; Chen et al., 2016). Exogenous EMAP-II could enhance the synergic effects of bortezomib with gemcitabine by cleaving caspase-3 in pancreatic cancer cells (Awasthi et al., 2013). However, whether EMAP-II enhances the cytotoxic effects of TMZ on GSCs remains unknown.

The process of mitochondrial turnover predominantly comprises autophagic sequestration and the delivery of mitochondria to lysosomes for hydrolytic degradation, a phenomenon known as mitophagy (Tolkovsky et al., 2002; Devin and Rigoulet, 2007). Mitophagy is a process in which damaged mitochondria are degraded to maintain cell metabolic homeostasis. However, mitophagy may also entail the clearance of healthy mitochondria in response to hypoxia and starvation, causing cellular metabolic dysfunction and even cell death (Kanki and Klionsky, 2009; Kanki et al., 2009; Kurihara et al., 2012). Bcl-2/adenovirus E1B 19 kDa protein-interacting protein 3 (BNIP3) is a member of the Bcl-2 subfamily of death-inducing proteins and has been found to interact with adenoviruses E1B-19K (Bellot et al., 2009). BNIP3 affects mitophagy by interacting with mitochondria with BNIP3 transmembrane domains (Zhang et al., 2008). It has been reported that TMZ can induce mitophagy in the U87 cell line (Lin et al., 2012), but the specific mechanism underlying this effect has not yet been elucidated. Whether TMZ exerts cytotoxic effect on GSCs though BNIP3-mediated mitophagy, as well as the mechanism though which TMZ exerts its effects, remain unclear.

MicroRNAs (miRNAs) are a family of endogenous, small (18–25 nucleotides) non-coding single-stranded RNA molecules found in all eukaryotic cells. Recent studies have shown that miRNAs can indirectly adjust autophagy, as well as mitophagy (Li et al., 2015). MiR-27a and miR-27b regulate autophagic clearance of damaged mitochondria by targeting PINK1 (Kim et al., 2016). MiR-24-3p has been found to be significantly overexpressed in oral squamous cell carcinoma (Lin et al., 2010) and promotes glioma cell proliferation (Xu et al., 2013). We found that EMAP-II induced upregulation of autophagy-related protein 5 (ATG5) and autophagy-related protein 7 (ATG7) to promote autophagy that killed glioma cells by down-regulating miR-20a(12). However, whether EMAP-II induces mitophagy in GSCs via miR-24-3p and enhances the cytotoxic effects of TMZ on GSCs has not been reported.

This study aims to verify whether low-dose EMAP-II enhances the cytotoxic effects of TMZ on GSCs and explores the mechanism by which EMAP-II induces BNIP3-dependent mitophagy via miR-24-3p down-regulation to enhance the inhibitory effects of TMZ on GSC malignant biological behavior.

## Materials and methods

### Cell lines and cell culture

The human malignant glioma cell lines U-87 and U-251 and the human embryonic kidney cell line HEK293T were purchased from the Shanghai Institutes for Biological Sciences Cell Resource Center. All the cells were cultivated in DMEM supplemented with 10% fetal bovine serum (FBS, Life Technologies Corporation, Paisley, UK), and all the cells were incubated in a moist incubator at 37°C with 5% CO_2_ and refreshed medium every 48 h.

### De-differentiation and identification of GSCs

GSCs were isolated as described previously (Tu et al., 2013). U87 and U251cells were trypsinized using 0.05% trypsin/EDTA and supplemented with DMEM/F-12 (Life Technologies Corporation, Grand Island, NY, USA), basic fibroblast growth factor (bFGF, 10 ng/ml, Life Technologies Corporation, Carlsbad, CA, USA), epidermal growth factor (EGF, 10 ng/ml, Life Technologies Corporation, Gaithersburg, MD, USA) and 2% B27 (50 ×, Life Technologies Corporation, Grand Island, NY, USA). Fresh media was added every third day of culture and the cells were passaged when average neurosphere size reached ~100–150 μm in diameter.

For differentiation experiments, spheres were dissociated by for a single cell suspension and seeded (2.5 × 104 cells/cm2) onto glass coverslips coated with poly-L-ornithine (BD Biosciences, Franklin Lakes, NJ, USA) in the medium without bFGF and EGF but containing 10 % FBS and were incubated for 7–11 days. For immunofluorescence staining of undifferentiated spheres, cells were incubated with antibodies against Nestin and CD133 (1:100, Santa Cruz Biotechnology, Santa Cruz, CA, USA). For immunostaining of differentiated spheres, cells were stained with antibodies against GFAP (1:100, Abcam, Cambridge, MA, USA) and beta-tubulin III (1:100, Santa Cruz Biotechnology). The primary antibody complexes were visualized with anti-rabbit Alexa Fluor 488 and anti-mouse Alexa Fluor 555 secondary antibodies (Beyotime Institute of Biotechnology, Jiangsu, China). Nuclei were counterstained with 4′, 6-diamidino-2-phenylindole (DAPI).

### Experimental groups

1. To investigate the effects of EMAP-II and TMZ on U87-GSCs and U251-GSCs, we divided the cells into the following four groups (*n* = *3*): (1) Control group, whose cells were treated with 0.9% sodium chloride (NS); (2) EMAP-II group, whose cells were treated with 0.05 nM EMAP-II for 0.5 h; (3) TMZ group, whose cells were treated with 400 μM TMZ for 48 h; and (4) EMAP-II+TMZ group, whose cells were treated with 400 μM TMZ for 48 h after pretreatment with 0.05 nM EMAP-II for 0.5 h.
2. To investigate whether BNIP3-mediated mitophagy was involved in regulating GSCs, we divided the cells into the following five groups (*n* = *3*): (1) Control group; (2) BNIP3(+)NC group, whose cells were transfected with a negative-control (NC) BNIP3-overexpressing plasmid; (3) BNIP3(+) group, whose cells were transfected with a BNIP3 overexpressing plasmid; (4) BNIP3(–)NC group, whose cells were transfected with an NC BNIP3-silencing plasmid; and (5) BNIP3(–) group, whose cells were transfected with a BNIP3-silencing plasmid.
3. To investigate whether BNIP3 participated in the effects exerted by the combination of EMAP-II and TMZ on GSCs, we divided the cells into the following four groups (*n* = *3*): (1) Control group; (2) EMAP-II+TMZ group, whose cells were treated with 400 μM TMZ for 48 h after pretreatment with 0.05 nM EMAP-II for 0.5 h; (3) BNIP3(+)+EMAP-II+TMZ group, whose BNIP3-overexpressing cells were treated with 400 μM TMZ for 48 h after pretreatment with 0.05 nM EMAP-II for 0.5 h; and (4) BNIP3(–)+EMAP-II+TMZ group, whose BNIP3-silenced cells were treated with 400 μM TMZ for 48 h after pretreatment with 0.05 nM EMAP-II for 0.5 h.
4. To study the effects of miR-24-3p on mitophagy in GSCs, we divided the cells into the following five groups (*n* = *3*): (1) Control group; (2) Agomir-24-3p-NC group, whose cells were transfected with an NC miR-24-3p agomir; (3) Agomir-24-3p group, whose cells were transfected with a miR-24-3p agomir; (4) Antagomir-24-3p-NC group, whose cells were transfected with an NC miR-24-3p antagomir; and (5) Antagomir-24-3p group, whose cells were transfected with a miR-24-3p antagomir.
5. To investigate whether whether miR-24-3p could play a role in regulating BNIP3 in the combination of EMAP-II and TMZ, we divided the cells into the following four groups (*n* = 3):(1) Control group; (2) EMAP-II+TMZ group, whose cells were treated with 400 μM TMZ for 48 h after pretreatment with 0.05 nM EMAP-II for 0.5 h; (3) agomir-24-3p+EMAP-II+TMZ group, whose agomir-24-3p cells were treated with 400 μM TMZ for 48 h after pretreatment with 0.05 nM EMAP-II for 0.5 h; and (4) antagomir-24-3p+EMAP-II+TMZ group, whose antagomir-24-3p cells were treated with 400 μM TMZ for 48 h after pretreatment with 0.05 nM EMAP-II for 0.5 h.
6. To study the effects of miR-24-3p and BNIP3 on mitophagy, we divided the cells into the following nine groups (*n* = *3*): (1) Control group; (2) Agomir-24-3p-NC+BNIP3(+) NC group, whose cells were transfected with an NC miR-24-3p agomir and a BNIP3-overexpressing plasmid; (3) Agomir-24-3p+BNIP3(+) group, whose cells were transfected with a miR-24-3p agomir and BNIP3 overexpressing plasmid; (4) Agomir-24-3p-NC+BNIP3(-) NC group, whose cells transfected with an NC miR-24-3p agomir and a BNIP3-silencing plasmid; (5) Agomir-24-3p+BNIP3(–) group, whose cells were transfected with a miR-24-3p agomir and BNIP3 silencing plasmid; (6) Antagomir−24-3p-NC+BNIP3(+)-NC group, whose cells were transfected with an NC miR-24-3p antagomir and a BNIP3 overexpressing plasmid; (7) Antagomir-24-3p+BNIP3(+) group, whose cells were transfected with a miR-24-3p antagomir and BNIP3 overexpressing plasmid; (8) Antagomir−24-3p-NC+BNIP3(–) group, whose cells were transfected with an NC miR-24-3p antagomir and a BNIP3-silencing plasmid; and (9) Antagomir-24-3p+BNIP3(–) group, whose cells were transfected with a miR-24-3p antagomir and BNIP3-silencing plasmid.

### Cell viability assay

Cell Counting Kit-8 (CCK8, Dojindo, Tokyo, Japan) was used for cell cytotoxicity and proliferation assay. Cells were seeded in 96-well plates at a density of 5,000 cells per well, and 10 μl of CCK8 was added into each well at 24, 48, 72 h after DMSO or TMZ treatment (0, 100, 200, 400, 600, 800, 1000, 1200 μM). As the cells incubated at 37°C for 4 h, the absorbance was measured at a wave length of 450 nm every 30 min.

### Cell migration and invasion assay

Cell migration and invasion was measured using a transwell permeable chamber (6.5 mm in diameter, 8 μm pore size, Corning Incorporate, Corning, NY, USA). Cells were suspended in 100 μl of serum-free medium and then added to the upper chamber (without Matrigel for cell migration assay) or seeded in a Matrigel-pre-coated upper chamber (for cell invasion assay), while 500 μl of medium with 10% FBS was added to the lower chamber. Then, the medium in the lower chamber was replaced with 500 μl of 10% FBS medium. After incubation for 24 h, the cells that had migrated to or invaded the bottom of the membrane were fixed with methanol/glacial acetic acid mixture for 30 min and then stained with 10% Giemsa. Then the pictures of stained cells were taken with an inverted microscope. And the cell number in three randomly fields were counted. Data were collected from repeated three independent experiments.

### Cell transfection

The human BNIP3 gene was ligated into a pGCMV/MCS/IRES/EGFP/Neo vector (GenePharma, Shanghai, China) to construct a BNIP3(+) plasmid, and the shRNA sequence against BNIP3 was ligated into a pGPU6/GFP/Neo vector. The U87 and U251 cell lines were transfected with BNIP3(+)-encoding plasmids or BNIP3 shRNA and designated BNIP3(+) cells or BNIP3(–) cells, respectively. Empty vectors were used for the negative controls (NCs). Opti-MEM and Lipofectamine 3000 reagent (Life Technologies Corporation, Carlsbad, CA, USA) were used according to the manufacturer's instructions. Transfection was performed at about 80% confluency of cells in 24-well plates with Opti-MEM® I and Lipofectamine 3,000 reagent. Stable cell lines were obtained through selection using 0.4 mg/ml geneticin (G418; Sigma-Aldrich, St Louis, MO, USA). Geneticin-resistant cells were obtained after 4 or 5 weeks. Geneticin-resistant GSCs were subsequently isolated from these cells.

The cells were transfected with a miR-24-3p agomir, miR-24-3p antagomir, or the appropriate NC (GenePharma, Shanghai, China) using Lipofectamine 3,000 reagent (Life Technologies Corporation), according to the manufacturer's instructions. After 6 h of transfection, the medium was removed and replaced with fresh medium. The cells were harvested after 48 h of transfection.

### Immunofluorescence staining

Cells were stained with LysoTracker Red or MitoTracker Red at a final concentration of 50 nM. Then, the cells were fixed in 4% paraformaldehyde for 30 min, blocked with 5% BSA for 2 h, and incubated with the primary antibody for microtubule-associated protein one light chain 3 (LC3, 1:150, Abcam, Cambridge, MA, USA, ab51520) at 4°C overnight. Then, the cells were washed and incubated with the appropriate secondary antibody before being counterstained with DAPI (Beyotime Institute of Biotechnology, Jiangsu, China) for 10 min and visualized with a confocal microscope.

### Luciferase reporter assays

For the luciferase reporter assays, HEK-293T cells were seeded into 96-well plates. These cells were co-transfected with a wild-type (Wt) or mutant (Mut) pmirGLO-BNIP3 reporter plasmid and an agomir-24-3p or agomir-24-3p-NC. After 48 h, luciferase activity was measured using Dual-luciferase Reporter Assay System (Promega, Madison, WI, USA). Relative firefly luciferase activity levels were calculated and normalized to that of renilla luciferase.

### RNA extraction and quantitative RT-PCR (qRT-PCR)

Total RNA was separated from U87-GSCs and U251-GSCs using Trizol reagent (Life Technologies Corporation). A Taq-Man MicroRNA Reverse Transcription Kit was used for miRNA reverse transcription (Applied Biosystems, Foster City, CA, USA). QRT-PCR was performed using TaqMan Universal Master Mix II with Taq-Man microRNA assays for miR-24-3p and U6 expression. For quantification of BNIP3 mRNA, we performed reverse transcription and RT-PCR amplification using an RT-PCR kit and SYBR Premix Dimer Eraser (TaKaRa, Dalian, China) and the assays for BNIP3 and GAPDH gene expression (Applied Biosystems). Fold changes were calculated using the relative quantification (2^−ΔΔCt^) method.

### Western blot analysis

U87-GSCs and U251-GSCs were lysed using ice-cold RIPA buffer and centrifuged at 17,000 × g at 4°C for 40 min. Protein concentrations were determined by a BCA Protein Assay Kit (Beyotime Institute of Biotechnology, Jiangsu, China). The proteins (30–60 μg) were separated by 10–15% SDS-PAGE and transferred onto PVDF membranes, which were blocked with 5% non-fat milk for 2 h and then incubated overnight with primary antibodies against LC3 (1:1000, Abcam, Cambridge, MA, USA, ab51520), P62/SQSTM1 (1:1000, Proteintech, Chicago, IL, USA, ab56416), GAPDH(1:10,000, Proteintech, Chicago, IL, USA, 6000-4-Ig), BNIP3 (1:1000, Abclonal, Cambridge, MA, USA, A5683), TOMM20 (1:1000, Proteintech, Chicago, IL, USA, 11802-1-AP), and CYPD (1:1000, Proteintech, Chicago, IL, USA, 12716-1-AP). After being washed with TBST, the membranes were incubated with the appropriate secondary antibodies. The protein bands were visualized with an enhanced chemiluminescence (ECL) kit (Santa Cruz Biotechnology) and scanned with ChemiImager 5500 V2.03 Software. The relative integrated density values (IDVs) were calculated using FluorChem 2.0 software and normalized to those of GAPDH.

### Subcutaneous and orthotopic xenografts in nude mice

Stably transfected U87-GSCs and U251-GSCs cells were used in the *in vivo* study. A lentivirus encoding a miR-24-3p silencer was generated using a pLenti6.3/V5eDEST Gateway Vector Kit (Life Technologies Corporation, Carlsbad, CA, USA). The miR-24-3p silencer was ligated into the pLenti6.3/V5eDEST vector (GenePharma, Shanghai, China), and then a pLenti6.3/V5eDEST-miR-24-3p-silencing vector was generated. The lentivirus was generated in 293FT cells using ViraPower Packaging Mix. After infection, the cells that stably expressed the miR-24-3p-silencer [miR-24-3p(–)] were selected.

Athymic nude mice (BALB/C-nu/nu, 4 weeks old, male) were purchased from the Cancer Institute of the Chinese Academy of Medical Science. The ethics committee of Shengjing Hospital has approved this study. The animals were raised in accordance with the guidelines of the Laboratory Animal Centre. The cells were subcutaneously implanted into the right flanks of the mice at a density of 5 × 10^5^ cells/mouse (*n* = 10 each group). Tumor volumes were measured every 5 days until 50 days post-inoculation and were calculated using the following formula: volume (mm^3^) = length × width^2^/2.

For intracranial orthotopic inoculation, 3 × 10^5^ cells were stereotactically implanted in the right striatum of the mice (*n* = 10 each group). Mice died within 7 days post-operation were considered to have unrelated to tumors and eliminated from the study. New mice were then treated and added to the groups. The numbers of surviving mice were recorded daily until 50 days post-operation. Survival analysis was performed using Kaplan-Meier survival curves. Tumor-bearing mice were divided into the following four groups: (1) Control group, whose mice treated with 0.9% sodium chloride; (2) EMAP-II+TMZ group, whose mice were treated with 400 μM TMZ after pretreatment with 0.05 nM EMAP-II for 0.5 h; (3) miR-24-3p(–) group, whose mice were treated with cells stably expressing miR-24-3p(–); (4) EMAP-II+TMZ+miR-24-3p(–) group, whose mice were treated with cells stably expressing miR-24-3p(–) and 400 μM TMZ after pretreatment with 0.05 nM EMAP-II for 0.5 h.

### Transmission electron microscopy (TEM)

The cells were fixed in 2.5% glutaraldehyde (electron microscopy grade) in phosphate-buffered saline (PBS) at 4°C for 2 h, dehydrated in an ethanol series and embedded in Epon resin. Ultrathin sections were then subjected to TEM analysis. Representative areas were chosen for ultrathin sectioning and viewed with a Philips EM 400 transmission electron microscope at an accelerating voltage of 80 kV, and digital images were obtained by an AMT imaging system (Advanced Microscopy Techniques Cor, Danvers, MA, USA).

### Statistical analysis

Data are presented as the mean ± standard deviation (SD) and were analyzed using SPSS19.0 (SPSS, IL, USA). Statistical analysis was performed using Student's *t*-test and one-way ANOVA. *P* < 0.05 was considered statistically significant. IC50 was measured by GraphPad Prism software (raw data in Data Sheet 1).

## Results

### De-differentiation and identification of GSCs

The result we already published showed that the isolated GSCs using sphere culture by flow cytometry analysis contained 98.2 and 98.6% CD133+ cells in GSCs-U251 and GSCs-U87, respectively (Zhang et al., 2013). To identify GSCs, we use immunofluorescence staining. The positive staining of Nestin and CD133 confirmed that most cells within the spheres expressed these neural stem cell lineage markers on their membranes (Supplementary Figure 1A). Besides, the cell spheres are differentiated into a non-GSC line in DMEM supplemented with 10% FBS, without EGF or FGF. These cells showed typical morphological differentiation toward neuronal and astrocytic lineages, identified as beta-tubulin-III positive neurons and GFAP-positive astrocytes (Supplementary Figure 1B).

### EMAP-II intensified the suppressive effects of TMZ on U87-GSCs and U251-GSCs viability, migration, and invasion

**Figure 1:**
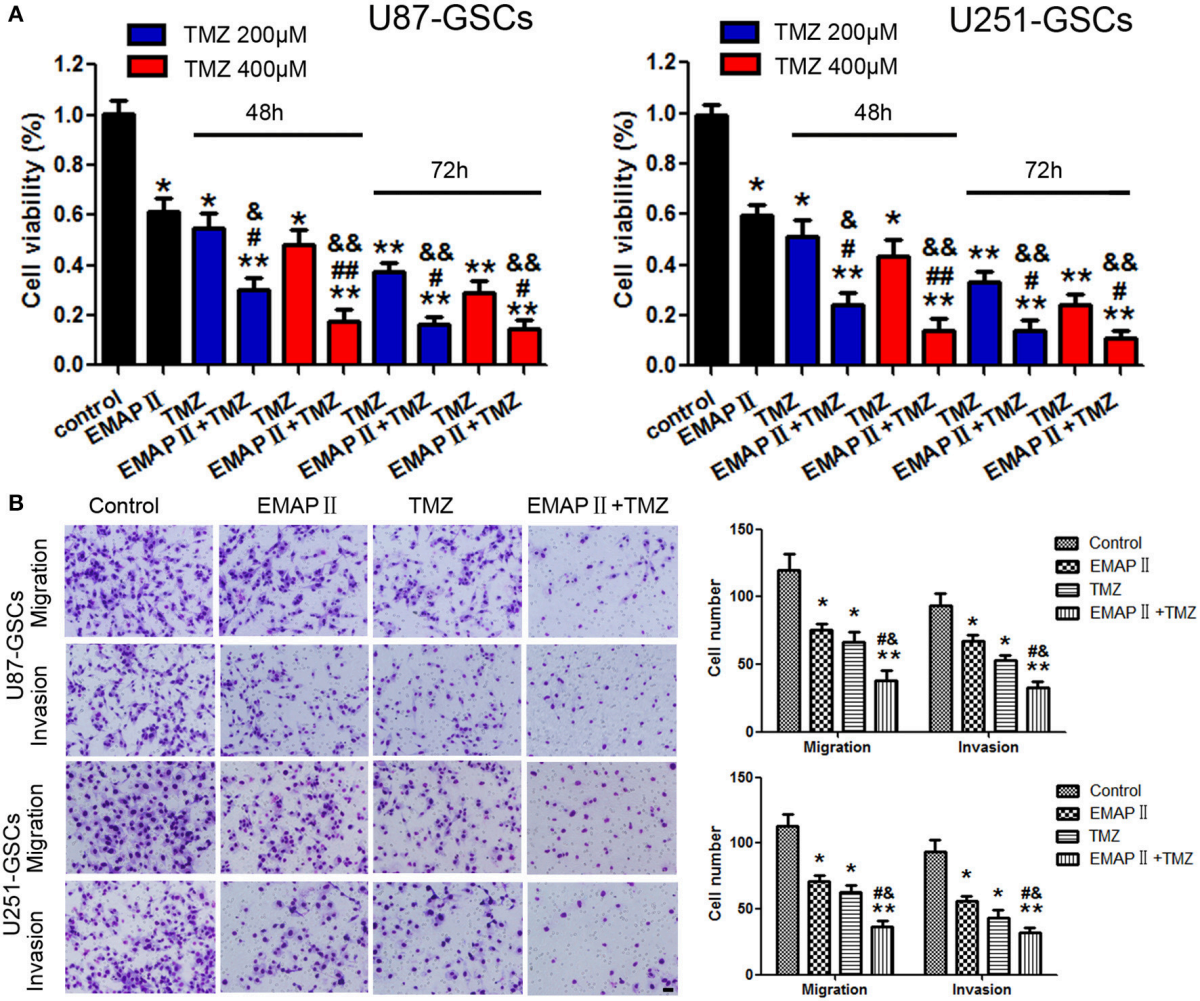
The effects of TMZ and EMAP-II on U87-GSCs and U251-GSCs viability, migration and invasion. **(A)** Cytotoxic effects of the combination of EMAP-II and TMZ on U87-GSCs and U251-GSCs compared with the control treatment and EMAP-II and TMZ alone. The combination of EMAP-II and TMZ significantly inhibited cell viability of U87-GSCs and U251-GSCs compared with the control treatment and EMAP-II and TMZ alone. Data are presented as the mean ± SD from three independent experiments. **(B)** GSC cell migration and invasion were investigated by transwell assays after treatment with EMAP-II and TMZ. Compared with the control treatment and EMAP-II and TMZ alone, the migration and invasion of GSC are inhibited in the combination of EMAP-II and TMZ group. Data are presented as the mean ± SD of *n* = 3. ^*^P < 0.05, ^**^P < 0.01 vs. control group, ^#^*P* < 0.05, ^##^*P* < 0.01 vs. TMZ group, ^&^*P* < 0.05, ^&&^*P* < 0.01 vs. EMAP-II group. Scale bars represent 40 μm.

The cytotoxicity of TMZ and EMAP-II was measured by Cell Counting Kit-8 (CCK-8).Based on the IC50 values of TMZ against U87-GSCs and U251-GSCs (Zhou et al., 2017), we selected the concentrations of 200 and 400 μM and the time periods of 48 or 72 h for subsequent experiments. According to the results of our preliminary study (Chen et al., 2016), 0.05 nM EMAP-II and 0.5 h were selected as the optimum pretreatment concentration and time point, respectively. Viability was apparently inhibited in the EMAP-II and TMZ groups and the EMAP-II and TMZ combination group after 48 and 72 h of treatment (*P* < 0.05, *P* < 0.05, *P* < 0.01). Compared with the EMAP-II and TMZ groups, the EMAP-II and 200 and 400 μM TMZ combination groups displayed significantly reduced cell viability after 48 h of treatment (*P* < 0.05, *P* < 0.01). The EMAP-II and 200 or 400 μM TMZ combination groups also displayed significantly lower cell viability than the EMAP-II group after 72 h of treatment (*P* < 0.05; Figure 1A). Thus, 0.05 nM EMAP-II for 0.5 h and 400 μM TMZ for 48 h were the pretreatment and treatment concentrations and times, respectively, used in subsequent experiments.

Next we explore the combination of EMAP-II and TMZ on migration and invasion of GSCs.U87-GSC and U251-GSC migration and invasion were significantly inhibited in the EMAP-II and TMZ groups and the EMAP-II and TMZ combination group (*P* < 0.05). Compared with the EMAP-II and TMZ groups, the EMAP-II and TMZ combination group displayed significantly decreased migration and invasion (*P* < 0.05; Figure 1B).

### Combination treatment with EMAP-II and TMZ induced mitophagy in GSCs

**Figure 2:**
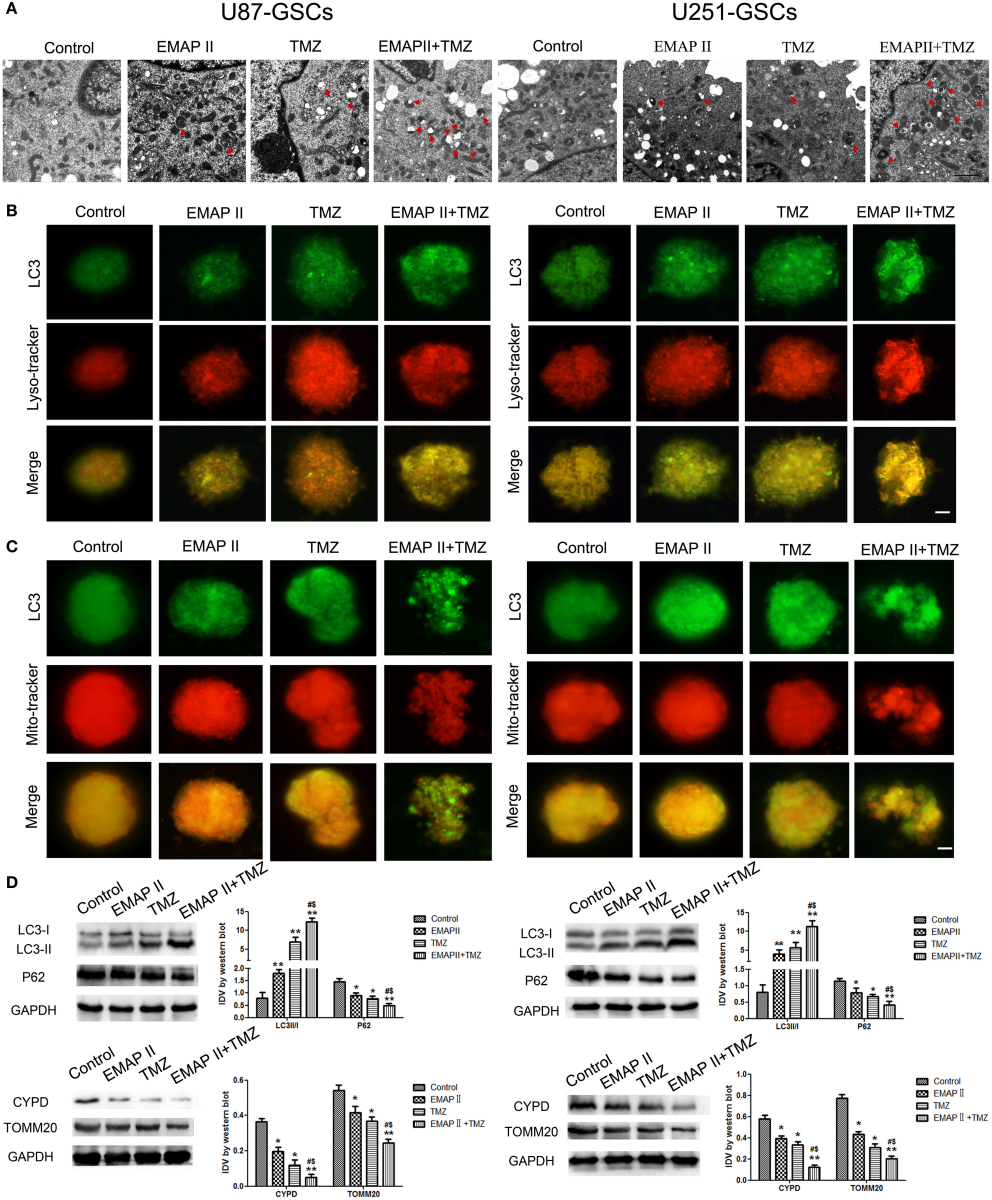
The effects of EMAP-II and TMZ on mitophagy in U87-GSCs and U251-GSCs. **(A)** Representative TEM image of autophagosomes in U87-GSCs and U251-GSCs treated with EMAP-II and TMZ. Autophagysome activity levels were gradually increased in the EMAP-II and TMZ groups and the EMAP-II and TMZ combination group. Scale bar corresponds to 1 μm. **(B,C)** The distribution and expression of LC3 in U87-GSCs and U251-GSCs treated with EMAP-II and TMZ were analyzed by immunofluorescence assay with Lyso-Tracker and Mito-Tracker. The expression of LC3 indicates the occurrence of autophagy. The distribution of LC3 that overlap with lysosome or mitochondria indicates autophagy or mitophagy. Scale bars represent 20 μm. **(D)** Western blot analysis of the LC3-II/I ratio and P62, TOMM 20, and CYPD expression levels in U87-GSCs and U251-GSCs treated with EMAP-II and TMZ. The LC3-II/I ratio increased, but P62, TOMM 20, and CYPD expression decreased in GSCs treated with EMAP-II and TMZ. Data are presented as the mean ± SD of *n* = 3. ^*^P < 0.05, ^**^P < 0.01 vs. control group, ^#^*P* < 0.05 vs. TMZ group, ^&^*P* < 0.05 vs. EMAP-II group.

TEM and Immunofluorescence assay was used to monitor mitophagy. Autophagysome activity levels were increased in the EMAP-II and TMZ groups and the EMAP-II and TMZ combination group. The EMAP-II and TMZ combination group displayed more autophagysomes than the EMAP-II and TMZ groups (Figure 2A). U87-GSC and U251-GSC were stained for LC3 (green) and Mito-Tracker or Lyso-Tracker (red). The punctate density and distribution indicates LC3 expression. LC3 punctuate staining was increased in the EMAP-II and TMZ groups and the EMAP-II and TMZ combination group. LC3 punctuate staining was dramatically increased in the EMAP-II and TMZ combination group compared with the EMAP-II and TMZ groups. Additionally, we noted an overlap between LC3 and Mito-Tracker or Lyso-Tracker. The latter two agents exhibited a staining trend similar to that of LC3, and their dots colocalized with the LC3 dots (Figures 2B,C).

Western blot was used to detect the protein expression of LC3 II/I, P62, mitochondrial matrix protein CYPD and mitochondrial membrane protein TOMM20 in U87-GSCs and U251-GSCs. The LC3-II/I ratio gradually increased in the EMAP-II and TMZ groups and the EMAP-II and TMZ combination group (*P* < 0.01), but P62, TOMM20 and CYPD expression levels displayed gradual decreases in the EMAP-II and TMZ groups and the EMAP-II and TMZ combination group (*P* < 0.05). The LC3-II/I ratio increased (*P* < 0.05), but P62, TOMM20 and CYPD expression levels decreased in the EMAP-II and TMZ combination group compared with the EMAP-II and TMZ groups (*P* < 0.05; Figure 2D).

### Combination treatment with EMAP-II and TMZ inhibited GSCs malignant behavior by upregulating BNIP3

**Figure 3:**
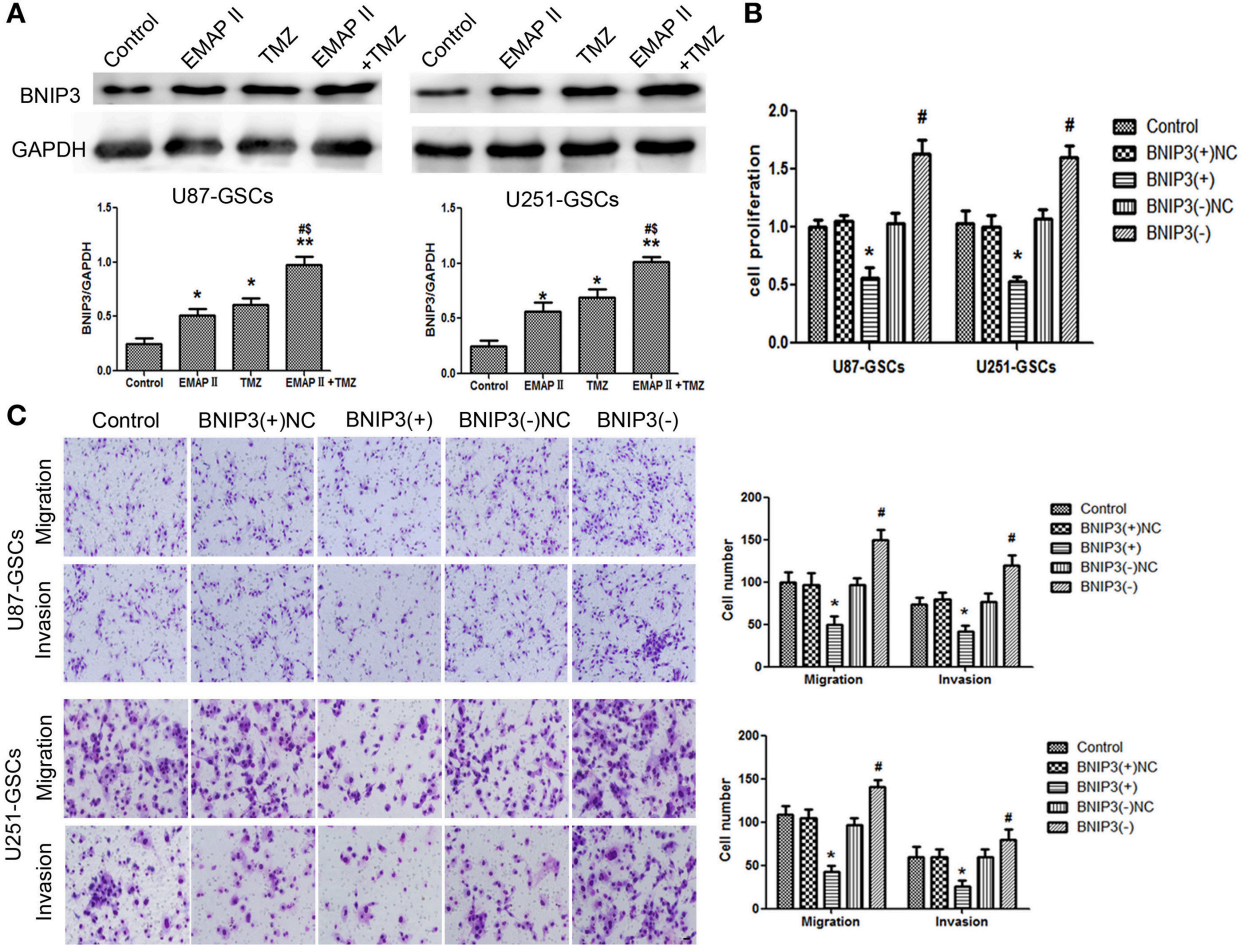
The effects of BNIP3 on GSC malignant behavior in U87-GSCs and U251-GSCs treated with EMAP-II and TMZ. **(A)** Effects of EMAP-II and TMZ on BNIP3 expression in U87-GSCs and U251-GSCs, as demonstrated by western blotting. The combination of EMAP-II and TMZ significantly increased the level of BNIP3 expression. Data are presented as the mean ± SD of *n* = 3. ^*^P < 0.05, ^**^P < 0.01 vs. control group, ^#^*P* < 0.05 vs. TMZ group, ^&^*P* < 0.05 vs. EMAP-II group. **(B)** The effects of BNIP3 on U87-GSC and U251-GSC proliferation were assessed by CCK-8. BNIP3 overexpression inhibited GSC proliferation. Data are presented as the mean ± SD of *n* = 3. ^*^P < 0.05 vs. BNIP3(+)-NC group, ^#^*P* < 0.05 vs. BNIP3(–)-NC group. **(C)** The effects of BNIP3 on U87-GSC and U251-GSC migration and invasion were assessed by transwell assays. BNIP3 overexpression inhibited GSC migration and invasion. Data are presented as the mean ± SD of *n* = 3. ^*^P < 0.05, vs. BNIP3(+)-NC group, ^#^*P* < 0.05 vs. BNIP3(–)-NC group.

EMAP-II and TMZ influenced the expression of BNIP3 in GSCs. BNIP3 protein expression increased significantly in the EMAP-II and TMZ groups and the EMAP-II and TMZ combination group (*P* < 0.05). BNIP3 protein expression increased in the EMAP-II and TMZ combination group compared with EMAP-II and TMZ groups (*P* < 0.05) (Figure 3A). Proliferation, migration, and invasion of GSCs were influenced by BNIP3 overexpression and knockdown. GSCs proliferation, migration, and invasion were inhibited in the BNIP3(+) group (*P* < 0.05) and were enhanced in the BNIP3(-) group (*P* < 0.05) (Figures 3B,C).

### Combination treatment of EMAP-II and TMZ induced mitophagy by up-regulating BNIP3

**Figure 4:**
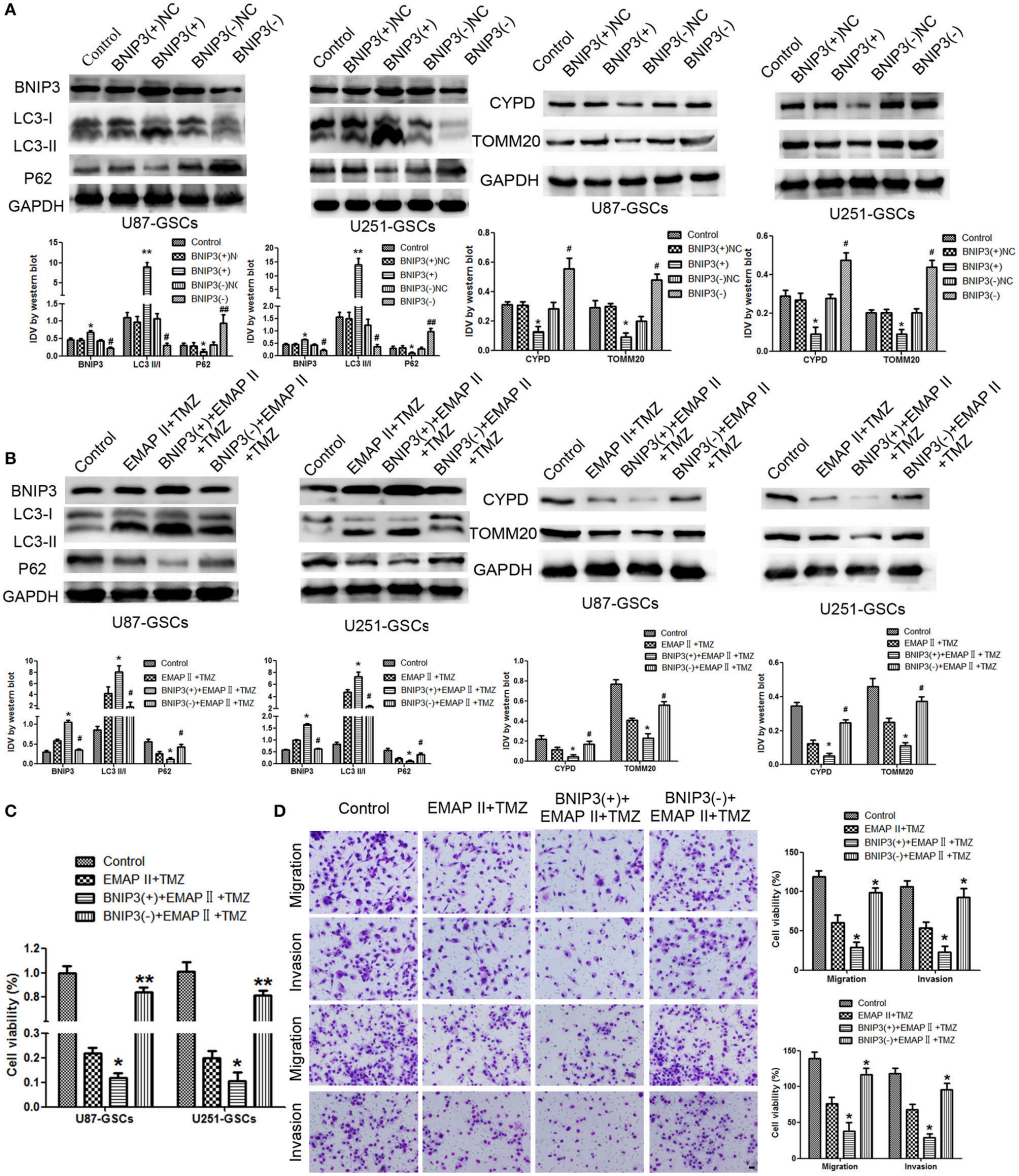
The effects of BNIP3 on mitophagy-related protein expression levels in U87-GSCs and U251-GSCs treated with EMAP-II and TMZ. **(A)** Western blot analysis of the effects of BNIP3 overexpression and silencing on the LC3-II/I ratio and BNIP3, P62, TOMM 20, and CYPD expression levels. BNIP3 overexpression up-regulated LC3-II/I ratio and reduced P62, TOMM 20, and CYPD expression. The Data are presented as the mean ± SD of *n* = 3. ^*^P < 0.05, ^**^P < 0.01 vs. BNIP3(+)-NC group, ^#^*P* < 0.05, ^##^*P* < 0.01 vs. BNIP3(–)-NC group. **(B)** Western blot analysis of the effects of BNIP3 overexpression and silencing and EMAP-II and TMZ on the LC3-II/I ratio and BNIP3, P62, TOMM 20, and CYPD expression levels. The LC3-II/I ratio significantly increased and P62, TOMM 20, and CYPD expression dramatically decreased in the combination of BNIP3 overexpression, EMAP-II and TMZ. Data are presented as the mean ± SD of *n* = 3. ^*^P < 0.05 vs. EMAP-II+TMZ group, ^#^*P* < 0.05 vs. EMAP-II+TMZ group.**(C,D)** CCK8 and transwell assays of the effects of BNIP3 overexpression or silencing and the combination of EMAP-II and TMZ on the proliferation, migration, and invasion of U87-GSCs and U251-GSCs. The combination of BNIP3 overexpression, EMAP-II and TMZ inhibited GSCs proliferation, migration, and invasion. Data are presented as the mean ± SD of *n* = 3. ^*^P < 0.05 vs. EMAP-II+TMZ group, ^#^*P* < 0.05, ^**^P < 0.01 vs. EMAP-II+TMZ group. Scale bars represent 40 μm.

We furtherly analyzed the effect of BNIP3 on mitophagy in GSCs. The LC3-II/I ratio and BNIP3 expression increased (*P* < 0.01, *P* < 0.05), but the expression levels of P62, TOMM20 and CYPD decreased in the BNIP3(+) group (*P* < 0.01, *P* < 0.05). Consistent with these findings, the LC3-II/I ratio and BNIP3 expression decreased (*P* < 0.01), and P62, TOMM20 and CYPD expression levels increased in the BNIP3(–) group (*P* < 0.01) (*P* < 0.05) (Figure 4A).

To explore whether BNIP3 displayed an important role in the combination of EMAP-II and TMZ, GSCs were treated with EMAP-II and TMZ based on whether BNIP3 was overexpressed or knocked down. The LC3II/I ratio and BNIP3 expression was increased (*P* < 0.05), but P62, TOMM20, and CYPD expression levels were decreased in the BNIP3(+)+EMAP-II+TMZ group compared with the EMAP-II and TMZ combination group (*P* < 0.05). The opposite result was observed in the BNIP3(–)+EMAP-II+TMZ group compared with the EMAP-II and TMZ combination group (Figure 4B). GSCs proliferation, migration and invasion were increased in the BNIP3(–)+EMAP-II+TMZ group compared with EMAP-II and TMZ combination group (*P* < 0.05) and were inhibited in the BNIP3(+)+EMAP-II+TMZ group compared with EMAP-II and TMZ combination group (*P* < 0.05; Figures 4C,D).

### Combination treatment with EMAP-II and TMZ induced mitophagy and inhibited GSC malignant behavior by down-regulating miR-24-3p

**Figure 5:**
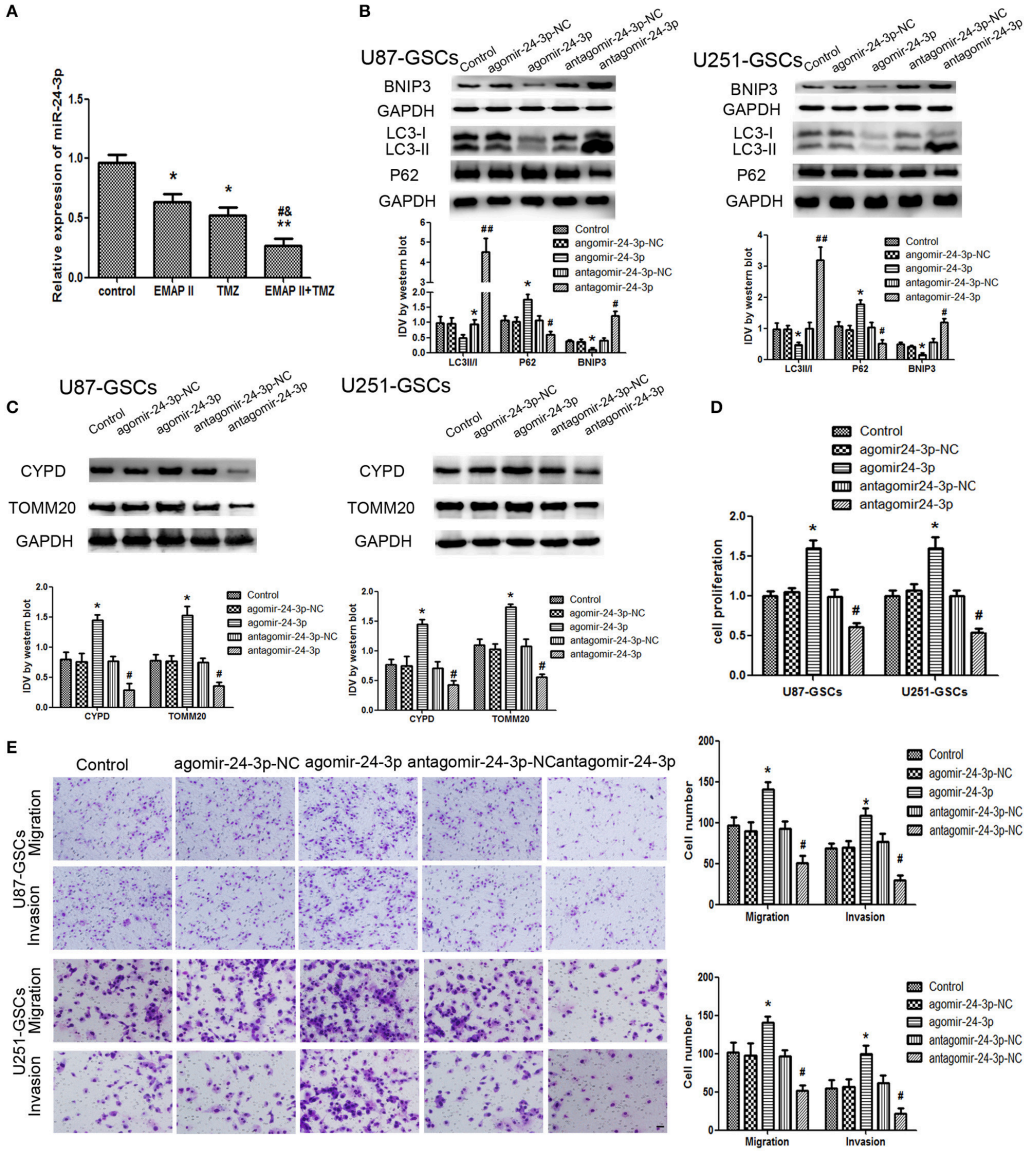
The effects of miR-24-3p on mitophagy-related protein expression levels and U87-GSC and U251-GSC malignant behavior. **(A)** Effects of EMAP-II and TMZ on MiR-24-3p expression, as demonstrated by qRT-PCR, in U87-GSCs and U251-GSCs. MiR-24-3p expression levels were obviously decreased in the EMAP-II and TMZ combination group. Data are presented as the mean ± SD of *n* = 3. ^*^P < 0.05, ^**^P < 0.01 vs. control group, ^#^*P* < 0.05 vs. TMZ group, ^&^*P* < 0.05 vs. EMAP-II group. **(B,C)** Western blot analysis of the effects of miR-24-3p overexpression and silencing on the LC3-II/I ratio and BNIP3, P62, TOMM 20, and CYPD expression levels. MiR-24-3p silencing up-regulated LC3-II/I ratio and BNIP3 expression and reduce P62, TOMM 20, and CYPD expression. Data are presented as the mean ± SD of *n* = 3. ^*^P < 0.05 vs. agomir-24-3p-NC group, ^#^*P* < 0.05 vs. antagomir-24-3p-NC group. **(D,E)** The effects of miR-24-3p on U87-GSC and U251-GSC proliferation, migration, and invasion were assessed by CCK-8 or transwell assays. MiR-24-3p silencing inhibited GSC proliferation, migration, and invasion. Data are presented as the mean ± SD of *n* = 3. ^*^P < 0.05 vs. agomir-24-3p-NC group, ^#^*P* < 0.05, ^##^*P* < 0.01 vs. antagomir-24-3p-NCgroup.

The expression level of miR-24-3p in GSCs was detected by real-time PCR. MiR-24-3p expression levels were decreased in the EMAP-II and TMZ groups and the EMAP-II and TMZ combination group (*P* < 0.05). MiR-24-3p expression levels were decreased in the EMAP-II and TMZ combination group compared with the EMAP-II and TMZ groups (*P* < 0.05; Figure 5A).

Mitophagy-related proteins were affected by miR-24-3p. The LC3-II/I ratio and BNIP3 protein expression were decreased (*P* < 0.05), but P62, TOMM20, and CYPD expression levels were increased in the Agomir-24-3p group compared with the corresponding Control group (*P* < 0.05). Conversion of LC3-I to LC3-II and BNIP3 protein expression were increased (*P* < 0.05), but P62, TOMM20, and CYPD expression levels were decreased in the Antagomir-24-3p group compared with the corresponding Control group (*P* < 0.05) (Figures 5B,C). GSC proliferation, migration and invasion were inhibited in the Antagomir-24-3p group compared with the corresponding Control group (*P* < 0.05) and were enhanced in the Agomir-24-3p group compared with the corresponding Control group (*P* < 0.05; Figures 5D,E).

### BNIP3 was the target gene of miR-24-3p

**Figure 6:**
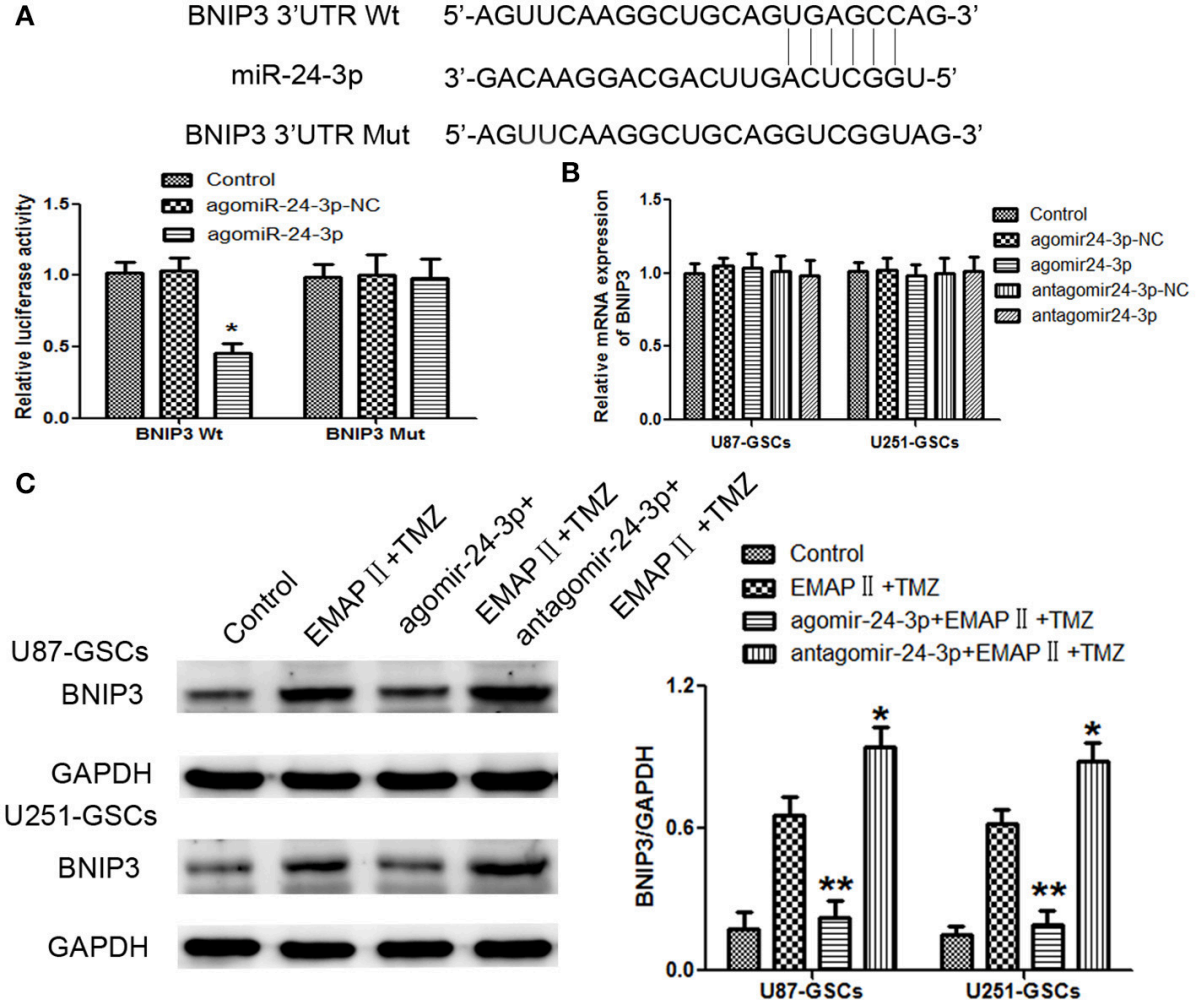
BNIP3 was the direct target of miR-24-3p, and its expression level changed after miR-24-3p overexpression or silencing in U87-GSCs and U251-GSCs. **(A)** Relative luciferase activity was expressed as firefly/renilla luciferase activity. Luciferase activity was significantly decreased in the Wt BNIP3 3′ untranslated region (3′UTR)+agomir-24-3p group. Data are presented as the mean ± SD of *n* = 3. ^*^P < 0.05 vs. agomir-24-3p-NC group. **(B)** BNIP3 relative mRNA expression levels were detected by qRT-PCR in U87-GSCs and U251-GSCs after miR-24-3p overexpression or silencing. MiR-24-3p overexpression or silencing do not influence BNIP3 mRNA expression. Data are presented as the mean ± SD of *n* = 3. **(C)** Western blot analysis of BNIP3 levels in U87-GSCs and U251-GSCs treated with EMAP-II and TMZ on the basis of miR-24-3p overexpression or silencing. Compared with EMAP-II+TMZ group, the expression of BNIP3 was reduced in agomir-24-3p+ EMAP-II+TMZ group. The opposite result was in antagomir-24-3p+EMAP-II+TMZ group. Data are presented as the mean ± SD of *n* = 3, ^**^P < 0.01 vs. EMAP-II+TMZ group, ^*^P < 0.05 vs. EMAP-II+TMZgroup.

By using TargetScan Human Release 6.2 Software, there was a potential binding site between miR-24-3p and 3′-UTR of BNIP3. Dual-luciferase gene reporter assay proved luciferase activity was significantly decreased in the Wt BNIP3 3′ untranslated region (3′UTR)+agomir-24-3p group (*P* < 0.05). There was no significant difference in luciferase activity between the Mut BNIP3 3′UTR+agomir-24-3p-NC group and Mut BNIP3 3′UTR+agomir-24-3p group (*P* > 0.05; Figure 6A).

BNIP3 mRNA expression levels were not significantly different between the treated groups and their respective control groups (*P* > 0.05) (Figure 6B).

To furtherly explore whether miR-24-3p could affect BNIP3 in the combination of EMAP-II and TMZ, GSCs were treated with EMAP-II and TMZ on the basis of over-express or down-regulate miR-24-3p. Compared with EMAP-II+TMZ group, the expression of BNIP3 was reduced in agomir-24-3p+ EMAP-II+TMZ group; the expression of BNIP3 was increased in antagomir-24-3p+EMAP-II+TMZ group (Figure 6C).

### BNIP3-mediated mitophagy influenced GSC malignant behavior by down-regulating miR-24-3p

**Figure 7:**
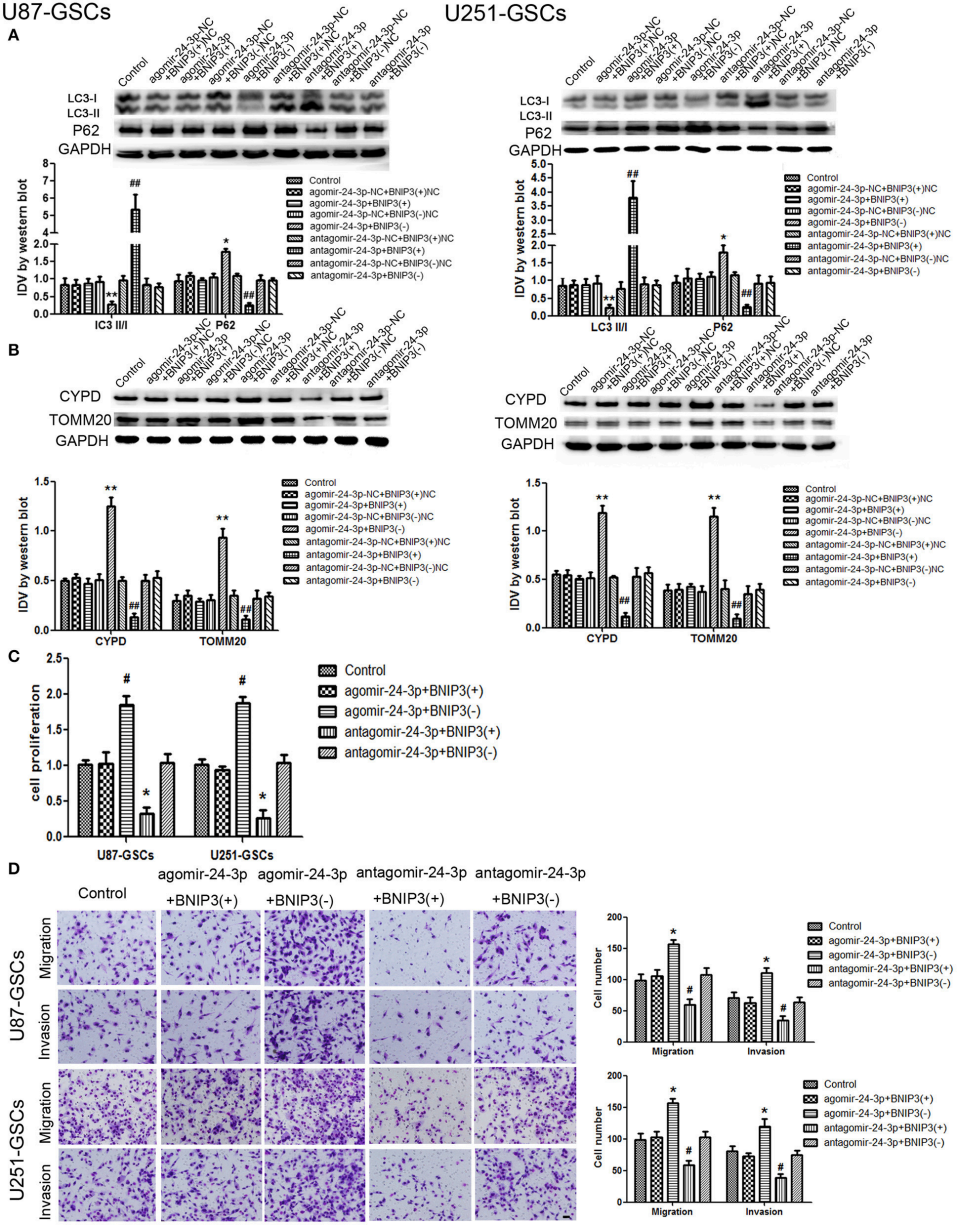
The effects of miR-24-3p and BNIP3 on mitophagy-related protein expression levels and U87-GSC and U251-GSC malignant behavior. **(A,B)** Western blot analysis of the effects of miR-24-3p and BNIP3 co-transfection on LC3-II/I, P62, TOMM 20, and CYPD expression levels. The LC3-II/I ratio was decreased, but P62, TOMM20 and CYPD expression levels were increased in the Agomir-24-3p+BNIP3(–) group. Data are presented as the mean ± SD of *n* = 3. ^*^P < 0.05, ^**^P < 0.01 vs. agomir-24-3p-NC+BNIP3(–)NC group, ^##^*P* < 0.01 vs. antagomir-24-3p-NC+ BNIP3(+)NC group. **(C,D)** The effects of miR-24-3p and BNIP3 on U87-GSC and U251-GSC proliferation, migration and invasion were assessed by CCK-8 or transwell assays. GSC proliferation, migration and invasion were significantly enhanced in the Agomir-24-3p+BNIP3(–) group. Data are presented as the mean ± SD of *n* = 3. ^*^P < 0.05 vs. control group, ^#^*P* < 0.05 vs. control group.

The expression of mitophagy-related proteins was affected by cotransfection of BNIP3 and miR-24-3p. No significant differences in LC3II/I, P62, TOMM20, and CYPD expression levels were detected in the Agomir-24-3p+BNIP3(+) and Antagomir-24-3p+BNIP3(–) groups compared with the corresponding Control groups (*P* >0 .05). The LC3-II/I ratio was decreased (*P* < 0.05), but P62, TOMM20 and CYPD expression levels were increased in the Agomir-24-3p+BNIP3(–) group (*P* < 0.05, *P* < 0.01, *P* < 0.01). Conversion of LC3-I to LC3-II was increased (*P* < 0.01), but P62, TOMM20, and CYPD expression levels were decreased in the Antagomir-24-3p+BNIP3(+) group (*P* < 0.01) (Figures 7A,B).

GSC proliferation, migration and invasion were enhanced in the Agomir-24-3p+BNIP3(–) group (*P* < 0.05). The opposite results were noted in the Antagomir-24-3p+BNIP3(+) group (*P* < 0.05) (Figures 7C,D).

### The combination of miR-24-3p, EMAP-II and TMZ inhibited tumor growth *in vivo* by inducing BNIP3-mediated mitophagy

**Figure 8:**
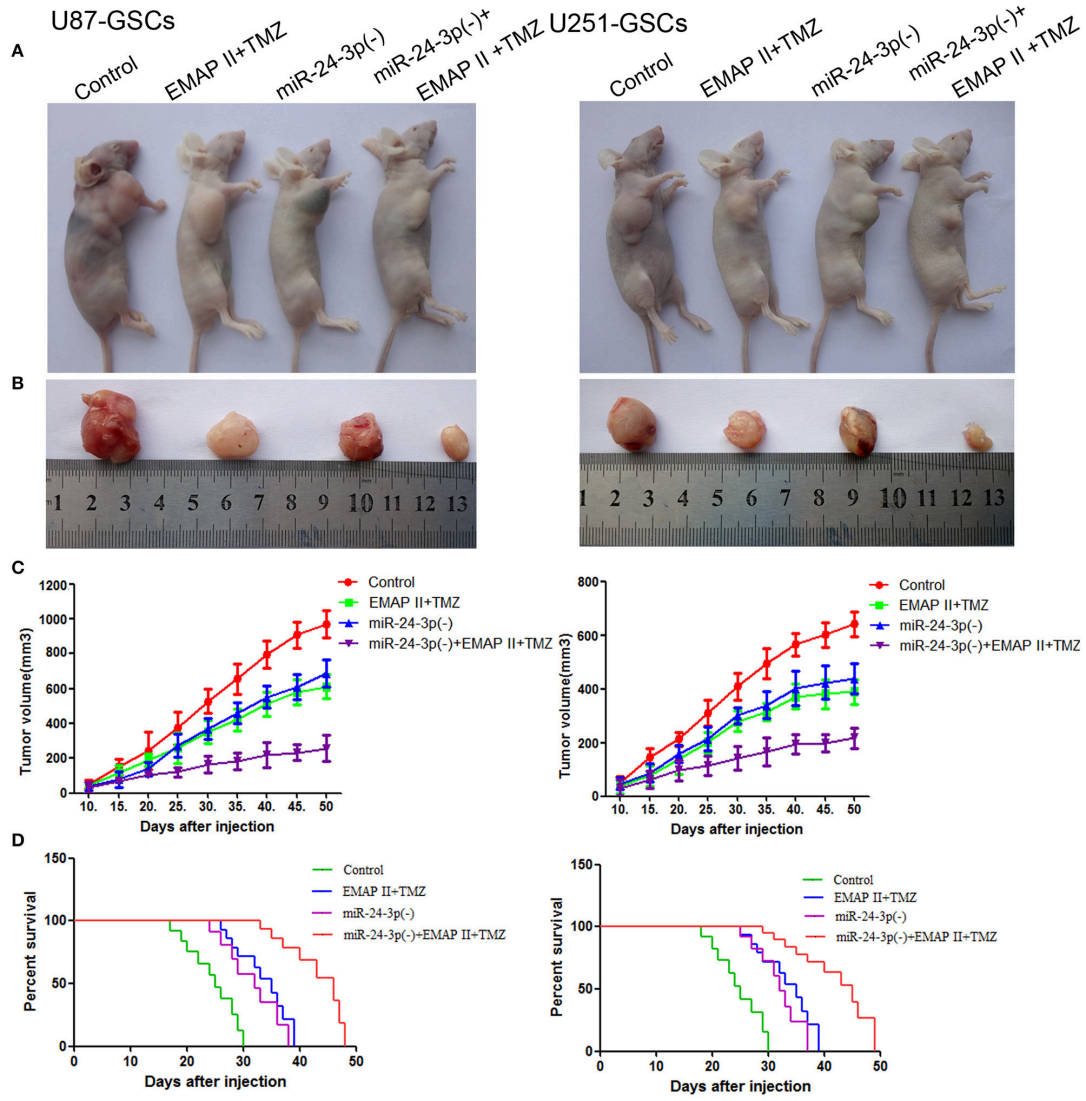
MiR-24-3p(–), EMAP-II and TMZ inhibited tumor growth *in vivo*. **(A–C)** Tumor growth curves in nude mice. Tumor volumes were calculated every 5 days after injection (*n* = 10). The minimum volume occured in the miR-24-3p(–)+EMAP-II+TMZ group. Tumor volumes were monitored for up to 50 days. **(D)** Nude mouse survival curves (*n* = 10). Mouse survival time was significantly longer in the miR-24-3p(–)+EMAP-II+TMZ group than in the other groups. The miR-24-3p(–) indicates miR-24-3p inhibition.

To prove the above findings, the growth-inhibitory effect of EMAP-II, TMZ and miR-24-3p inhibitor on U87-GSCs and U251-GSCs was tested in xenografted mice. The tumor volumes of the xenografts were significantly smaller in the EMAP-II+TMZ, miR-24-3p(–) and miR-24-3p(–)+EMAP-II+TMZ groups than in the Control group. The tumor volume of EMAP-II+TMZ group is smaller than that of the EMAP-II and the TMZ group. The minimum volume was noted in the miR-24-3p(–)+EMAP-II+TMZ group (Figures 8A–C). Mouse survival time were much longer in the EMAP-II+TMZ, miR-24-3p(–) and miR-24-3p(–)+EMAP-II+TMZ groups than in the Control group. Mouse survival time was significantly longer in the miR-24-3p(–)+EMAP-II+TMZ group than in the other groups (Figure 8D).

In addition, we explore the molecular changes in tumor tissues. Compared with control group, The LC3-II/I ratio and BNIP3 expression was increased (*P* < 0.05, *P* < 0.05), but P62, TOMM20 and CYPD expression levels were decreased in the EMAP-II+TMZ, miR-24-3p(–) and miR-24-3p(–)+EMAP-II+TMZ groups [*P* < 0.05, *P* < 0.05, *P* < 0.05).Compared with the EMAP-II+TMZ, miR-24-3p(–) group, The LC3-II/I ratio and BNIP3 expression was increased (*P* < 0.05, *P* < 0.05)], but P62, TOMM20 and CYPD expression levels were decreased in miR-24-3p(–)+EMAP-II+TMZ group(*P* < 0.05, *P* < 0.05, *P* < 0.05). All the results show that miR-24-3p-BNIP3- mitophagy is related with xenografted tumor growth. (Supplementary Figure 2).

## Discussion

We found that EMAP-II enhanced the cytotoxic effects of TMZ on GSC viability, migration and invasion. The combination of TMZ and EMAP-II induced GSC mitophagy, which was shown to inhibit GSC malignant behavior by upregulating BNIP3-mediated mitophagy. We also found that the combinaltion of TMZ and EMAP-II decreased miR-24-3p expression and that BNIP3 was the target gene of miR-24-3p. The combination of TMZ and EMAP-II induced mitophagy to inhibit malignant GSC behavior by down-regulating miR-24-3p. The results of our experiments with the xenografted mice showed that the EMAP-II+TMZ, miR-24-3p(–) and miR-24-3p(–)+EMAP-II+TMZ groups had smaller tumors and longer survival times than the other groups. In particular, the miR-24-3p(−)+EMAP-II+TMZ group had the smallest tumors and the longest survival time.

TMZ resistance is one of the major problems in chemotherapy. Some potential mechanisms underlying this resistance have been reported, including O6-methylguanine-DNA-methyltransferase (*MGMT*) expression, GSC microenvironment-induced resistance and autophagy-induced resistance (Kanzawa et al., 2004; Singh et al., 2004; Ohka et al., 2012). GSCs are less sensitive to chemotherapy than glioma cells, which is one of the reasons why the above chemotherapy has only limited effectiveness in those cells (Yu et al., 2015). In our previous study which has different mechanism with our recent study (Zhou et al., 2017), we found that GSC viability was affected by TMZ in a time-dependent manner over 24, 48, and 72 h of treatment. In addition, we found that the suppressive effects of TMZ on GSCs were also dose-dependent. However, when the TMZ concentration was 400 μM or higher, this dose-dependent effect was no longer observed, a finding consistent with those of other studies. Yu et al. found that the cytotoxic effects of TMZ on U251-GSCs and U87-GSCs were time- and dose-dependent and that 200 μM TMZ treatment for 48 h could decrease cell viability by almost 30% *(*Yu et al., 2015*)*. In recent years, studies have shown that EMAP-II plays an anti-tumor role through several processes, as the agent inhibits angiogenesis, promotes endothelial cell apoptosis and directly promotes tumor cell autophagy and apoptosis (Crippa et al., 2008; Li Z. et al., 2014; Ma et al., 2015; Chen et al., 2016). EMAP-II is still in the experimental study stage at present. Some studies have found that EMAP-II could facilitate TNF-R1 apoptotic signaling, which exerts anti-tumor functions (van Horssen et al., 2006). It also increases the permeability of blood-tumor barrier. Both are of benefit to killing tumor cells. Therefore, we think that EMAP-II has a good applied future in the clinical practice. However, whether EMAP-II can enhance the cytotoxicity of TMZ remains unclear. It has been reported that low-dose EMAP-II generated cytotoxic effects through the PI3K/Akt/FoxO1 axis in U87-GSCs *(*Liu et al., 2014*)*. TMZ could activate the Akt/glycogen synthase kinase-3ß pathway to induce glioma cell apoptosis via oncoprotein c-Myc (De Salvo et al., 2011) and exerted anti-tumor effects through the AKT-mTOR pathway in the GBM8901 glioma cell line (Chen et al., 2015). EMAP-II and TMZ affected glioma cells through a similar pathway, indicating that EMAP-II may strengthen the cytotoxic effects of TMZ. Our team have proved that the combination of EMAP-II and TMZ kill GSCs by inducing autophagy. Autophagy inhibitors weaken the cytotoxic effect of EMAP-II and TMZ on GSCs. Our research results are consistent with previous studies(Zhou et al., 2017). We also found that EMAP-II and TMZ kill GSCs through inducing mitophagy. We found that the cytotoxic effects of TMZ on GSCs were significantly enhanced and that GSC migration and invasion were also inhibited after EMAP-II pretreatment, but the concrete mechanisms underlying these effects remain to be investigated further.

Mitophagy plays an important role in a variety of diseases, such as diabetes, cancer and degenerative diseases of the nervous system (Greene et al., 2003; Soleimanpour et al., 2015; Fader et al., 2016; Wilfinger et al., 2016). Consequently, mitophagy has been regarded as a target in anti-tumor therapy. Recently, some chemotherapy drugs were also found to exert anti-tumor effects by inducing mitophagy. For example, salinomycin promoted mitophagy to inhibit the growth of breast cancer and prostate cancer cells (Jangamreddy et al., 2013), and sodium selenite induced superoxide-mediated mitophagy and subsequent autophagic cell death in malignant glioma cells (Kim et al., 2007). EMAP-II can induce mitophagy in the U87, U118 and GSCs cell lines (Ma et al., 2015), but the specific mechanism underlying this effect remains unclear. TMZ has been found to induce mitochondrial-mediated autophagy and reduce the quantity of mitochondria in the U87 cell line (Lin et al., 2012). Our results showed the combination of EMAP-II and TMZ promoted the formation of autophagosome by transmission electron microscopy and increased the overlap between LC3 and Mito-Tracker or Lyso-Tracker by immunofluorescence staining. EMAP-II and TMZ up-regulated LC3II/I ratio and BNIP3 expression but down-regulated P62, TOMM20, and CYPD expression, indicating promoting mitophagy. These results indicated that the combination of EMAP-II and TMZ inhibited GSC malignant behavior by enhancing mitophagy.

BNIP3 is distributed in the mitochondrial outer membrane and interacts with LC3 through the LC3-interacting region (LIR) to trigger excessive mitophagy (Hanna et al., 2012; Zhu et al., 2013). Arsenic trioxide and ceramide can upregulate BNIP3 expression to induce mitophagy in glioma (Daido et al., 2004; Kanzawa et al., 2005). We confirmed that the combination of EMAP-II and TMZ inhibited the biological behavior of GSCs by upregulated BNIP3 expression. BNIP3 overexpression enhanced the effect of EMAP-II and TMZ, which up-regulated the LC3II and down-regulated the expression level of P62, TOMM20 and CYPD, and inhibited the biological behavior of GSCs. BNIP3 knockdown exerted the opposite effect. The results of the above demonstrated the combination of EMAP-II and TMZ induced mitophagy by upregulating BNIP3 expression. BNIP3 overexpression could directly enhance the synergistic anti-tumor effects of EMAP-II and TMZ by inducing mitophagy. However, BNIP3 knockdown exerted the opposite effects. These results indicated that BNIP3-mediated mitophagy is one of the anti-tumor mechanisms underlying the effects of the combination of EMAP-II and TMZ. Abnormal increases in BNIP3 expression are affected by HIF-1 expression, which is positively correlated with tumor metastasis in non-small cell lung cancer (Giatromanolaki et al., 2004). BNIP3 silencing caused by promoter methylation and histone acetylation is associated with chemotherapy resistance and worse survival, and BNIP3 restoration enhances chemosensitivity and promotes tumor cell apoptosis in gastric and colon cancer (Murai et al., 2005). These results indicates BNIP3 loss caused by various reasons is related with tumor progression, which consists with our results.

MiRNAs plays key roles in tumourigenesis and tumor progression as oncomirs (oncogenic miRNAs) or tumor suppressors. EMAP-II upregulated ATG7 and ATG5 to enhance U87 and U251 glioma cell autophagy via miR20a down-regulation *(*Chen et al., 2016*)*. It has also been reported that TMZ upregulated ATG7 expression to induce autophagy by down-regulating miR-17 (Comincini et al., 2013). Previous study show miR-24-3p promoted cell proliferation and inhibited apoptosis in human breast cancer cells by targeting p27Kip1 (Lu et al., 2015). Moreover, miR-24-3p promoted cell proliferation in glioma cells via cooperative regulation of MXI1 (Xu et al., 2013). The results of these studies suggested that miR-24-3p may serve as an oncomir. And can EMAP-II and TMZ regulated mitophagy via miR-24-3p? Our results showed that the combination of EMAP-II and TMZ reduced miR-24-3p expression. MiR-24-3p overexpression decreased the LC3-II/I ratio and increased P62, TOMM20, and CYPD expression levels. MiR-24-3p overexpression also promoted the abilities of GSC proliferation, migration, and invasion. While miR-24-3p knockdown exerts the opposite effects. All the results indicated that combination treatment with EMAP-II and TMZ induced mitophagy and inhibited GSC malignant behavior by down-regulating miR-24-3p.

MiRNAs can bind to the 3′-UTRs of target mRNAs and block their translation. MiR-137 inhibited mitophagy by regulating the mitophagy receptors FUNDC1 and NIX (Li W. et al., 2014). BNIP3 was found to be the target gene of miR-24-3p using bioinformatics software. Dual-luciferase reporter assay illustrated that miR-24-3p binds with the 3′-UTR of BNIP3 mRNA. BNIP3 overexpression reversed the effects of agomir-24-3p on the LC3 II/I ratio and P62, TOMM 20, and CYPD expression, and BNIP3 knockdown reversed the effects of antagomir-24-3p on the LC3 II/I ratio and P62, TOMM 20, and CYPD expression. These resultsillustrate that miR-24-3p negatively regulates BNIP3, which inhibits mitophagy and promotes GSC proliferation, migration and invasion. Furthermore, we also found that combination of miR-24-3p(–), EMAP-II and TMZ inhibited tumor growth *in vivo*.

In summary, we have provided strong experimental evidence that EMAP-II can enhance the inhibitory effects of TMZ on GSC viability, migration and invasion. Regarding the mechanisms underlying this effect, the combination of TMZ and EMAP-II upregulated BNIP3 expression by down-regulating miR-24-3p, thereby strengthening BNIP3-mediated mitophagy. Because of its anti-tumor effects, the combination of EMAP-II and TMZ may represent a novel therapeutic strategy for treating glioma.

## Ethics statement

The animals were raised in accordance with the guidelines of the Laboratory Animal Centre of Shengjing Hospital.

## Author contributions

LL and YX: Conceived and designed the study; JZ, LL, and YX: Developed the methodology and performed the *in vitro* and *in vivo* experiments; YM and YL: Reviewed and interpreted the data; ZheL and ZhiL: Analyzed the data and supervised the study; JZ: Wrote the manuscript. All authors read and approved the final manuscript.

### Conflict of interest statement

The authors declare that the research was conducted in the absence of any commercial or financial relationships that could be construed as a potential conflict of interest.

## Supplementary material

Supplementary Figure 1 Isolation and identification of GSCs. **(A)** GSCs-U87 and GSCs-U251 stained for Nestin (red) andCD133 (green) by immunofluorescence staining. **(B)** GSC-U87 and GSC-U251 spheres were differentiated and then stained for GFAP (red) and beta-tubulin III (green) by immunofluorescence staining.

Supplementary Figure 2 Western blot analysis of the LC3-II/I ratio and BNIP3, P62, TOMM 20, and CYPD expression levels in U87-GSCs and U251-GSCs xenografted tumor. The LC3-II/I ratio and BNIP3 increased, but P62, TOMM 20, and CYPD expression decreased in the miR-24-3p(–)+EMAP-II+TMZ group. Data are presented as the mean ± SD of *n* = 3. ^*^*P* < 0.05 vs. control group, ^#^*P* < 0.05 vs. miR-24-3p(–) group, ^$^*P* < 0.05 vs. EMAP-II+TMZ group. The miR-24-3p(–) indicates miR-24-3p inhibition.

## Abbreviations

EMAP-II: endothelial-monocyte-activating polypeptide-II
TMZ: temozolomide
GSCs: glioma stem cells
EORTC/NCIC: European Organization for Research and Treatment of Cancer/National Cancer Information Center
MITC: 5-(3-methyltriazen-1yl) imidazole-4-carboxamide
BNIP3: Bcl-2/adenovirus E1B 19 kDa protein-interacting protein 3
ATG5: autophagy-related protein 5
ATG7: autophagy-related protein 7
EGF: epidermal growth factor
bFGF: basic fibroblast growth factor
FBS: fetal bovine serum
IDVs: integrated density values
NCCN: National Comprehensive Cancer Network.
