# Supplementary figures and images for: Endothelial Monocyte-Activating Polypeptide-II Induces BNIP3-Mediated Mitophagy to Enhance Temozolomide Cytotoxicity of Glioma Stem Cells via Down-Regulating MiR-24-3p
Source: Front Mol Neurosci. 2018 Mar 26;11:92. doi: 10.3389/fnmol.2018.00092 (PMC5879952; doi:10.3389/fnmol.2018.00092)

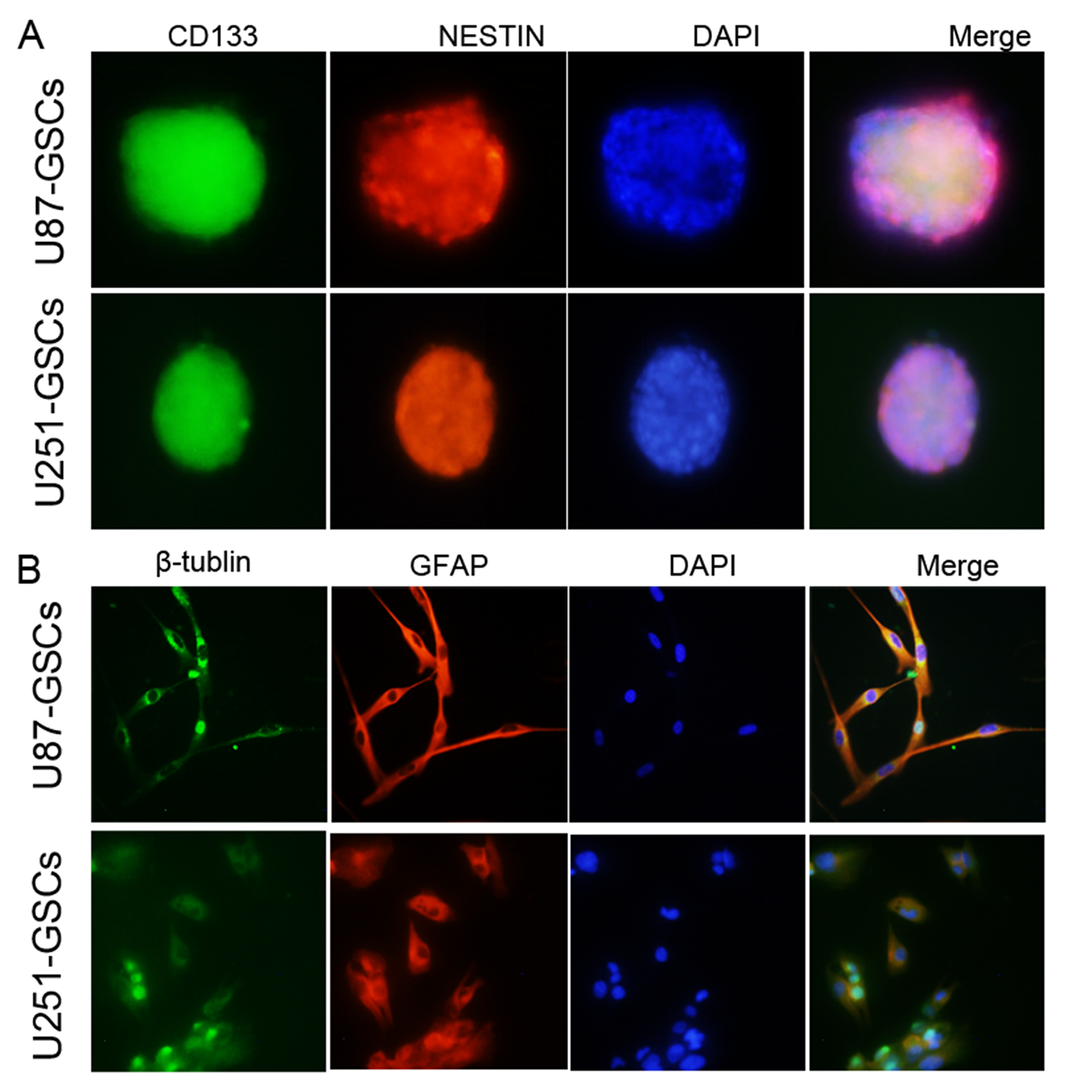

Supplement: Supplementary Figure 1 — Isolation and identification of GSCs. (A) GSCs-U87 and GSCs-U251 stained for Nestin (red) andCD133 (green) by immunofluorescence staining. (B) GSC-U87 and GSC-U251 spheres were differentiated and then stained for GFAP (red) and beta-tubulin III (green) by immunofluorescence staining. [file Image1.TIF]

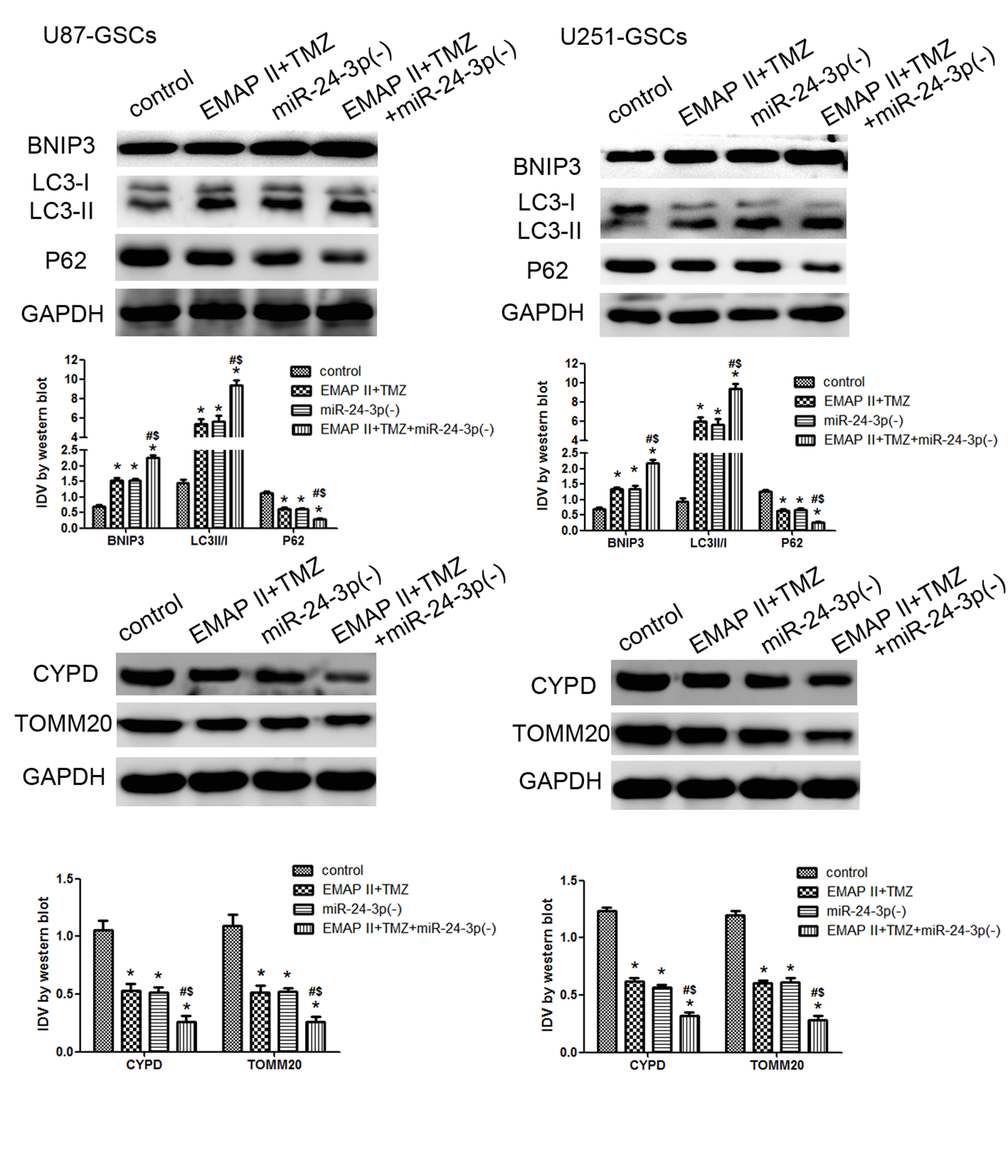

Supplement: Supplementary Figure 2 — Western blot analysis of the LC3-II/I ratio and BNIP3, P62, TOMM 20, and CYPD expression levels in U87-GSCs and U251-GSCs xenografted tumor. The LC3-II/I ratio and BNIP3 increased, but P62, TOMM 20, and CYPD expression decreased in the miR-24-3p(–)+EMAP-II+TMZ group. Data are presented as the mean ± SD of n = 3. *P < 0.05 vs. control group, #P < 0.05 vs. miR-24-3p(–) group, $P < 0.05 vs. EMAP-II+TMZ group. The miR-24-3p(–) indicates miR-24-3p inhibition. [file Image2.TIF]
